# Pressure and temperature predictions of Al_2_O_3_/water nanofluid flow in a porous pipe for different nanoparticles volume fractions: combination of CFD and ACOFIS

**DOI:** 10.1038/s41598-020-79689-x

**Published:** 2021-01-08

**Authors:** Meisam Babanezhad, Iman Behroyan, Azam Marjani, Saeed Shirazian

**Affiliations:** 1 Institute of Research and Development, Duy Tan University, Da Nang, 550000 Viet Nam; 2 Faculty of Electrical-Electronic Engineering, Duy Tan University, Da Nang, 550000 Viet Nam; 3 Department of Artificial Intelligence, Shunderman Industrial Strategy Co., Tehran, Iran; 4 Faculty of Mechanical and Energy Engineering, Shahid Beheshti University, Tehran, Iran; 5 Department of Computational Fluid Dynamics, Shunderman Industrial Strategy Co., Tehran, Iran; 6 Department for Management of Science and Technology Development, Ton Duc Thang University, Ho Chi Minh City, Viet Nam; 7 Faculty of Applied Sciences, Ton Duc Thang University, Ho Chi Minh City, Viet Nam; 8 Laboratory of Computational Modeling of Drugs, South Ural State University, 76 Lenin prospekt, 454080 Chelyabinsk, Russia

**Keywords:** Engineering, Mathematics and computing, Nanoscience and technology, Physics

## Abstract

Artificial intelligence (AI) techniques have illustrated significant roles in finding general patterns of CFD (Computational fluid dynamics) results. This study is conducted to develop combination of the ant colony optimization (ACO) algorithm with the fuzzy inference system (ACOFIS) for learning the CFD results of a physical case study. This binary join of the ACOFIS and CFD was used for pressure and temperature predictions of Al_2_O_3_/water nanofluid flow in a heated porous pipe. The intelligence of ACOFIS is investigated for different input numbers and pheromone effects, as the ant colony tuning parameter. The results showed that the intelligence of the ACOFIS could be found for three inputs (x and y nodes coordinates and nanoparticles fraction) and the pheromone effect of 0.1. At the system intelligence, the ACOFIS could predict the pressure and temperature of the nanofluid on any values of the nanoparticles fraction between 0.5 and 2%. Comparing the ANFIS and the ACOFIS, it was shown that both methods could reach the same accuracy in predictions of the nanofluid pressure and temperature. The root mean square error (RMSE) of the ACOFIS (~ 1.3) was a little more than that of the ANFIS (~ 0.03), while the total process time of the ANFIS (~ 213 s) was a bit more than that of the ACOFIS (~ 198 s). The AI algorithms process time (less than 4 min) shows their ability in the reduction of CFD modeling calculations and expenses.

## Introduction

Nanofluids possess an increasing utilization in thermal engineering industries in order to improve the heat transfer efficiency and reduce heat loss and energy costs^1–6^. In these novel heat transfer fluids (HTFs), the well-known Brownian movement of the nanoparticles (NPs) in the medium, known as the important parameter, influences the nanofluids' heat transfer rate^7^. Using porous media could also increase heat transfer rates considerably. The porous matrix causes more heat transfer area, higher thermal conductivity, and more mixing effect. So, it is a promising research area to use both nanofluid and porous media for different kinds of application such as heat exchangers (e.g. shell and tube type) due to the synergistic effect of nanofluids and porous media^8,9^.

Some studies have been focused on the nanofluids’ heat transfer in porous structures^10–12^. Nazari et al.^10^ experimentally analyzed heat transfer of water/CuO nanofluid (NF) in foam tubes with various types of configurations, and assessed the heat transfer rates^13^. It was revealed by them the improvements of heat transfer by the dispersion of the NPs into the base fluids for metal and helical foam tubes, comparing to the straight tube. In a study^12^, the development of convection for alumina/water NF was considered in a tube occupied by an aluminum metal foam. It was reported the heat transfer enhancement is caused by the increase of the nanofluid concentration and the Reynolds number as well.

As discussed above, a few studies have considered the potential of the application of nanofluids in porous ducts. So, this concept is still attractive for researchers in many aspects, specifically for process engineers to develop novel HTFs for industrial applications. Besides, the computational fluid dynamics (CFD) approach is a perfect and versatile tool for the predictions and simulations of thermal and hydrodynamic parameters of fluid flows in different regimes and geometries^14^. CFD modeling requires to solve many calculations on the system nodes to obtain the desired distributions, such as pressure and velocity. However, these calculations could be complex for 3-dimension analysis, turbulent flows, complex geometries, large scale cases, multi-phase flows, etc. For instance, the CFD modeling has its own difficulties and challenges for the evaluation of nanofluids flow characteristics in porous media. Changing in the nanoparticles concentrations is caused to the changes in thermo-physical properties of the nanofluids which need to be considered in the CFD simulations. Hence, a new simulation strategy is required for the new nanoparticles concentrations that can obviate the need for expensive and tedious CFD calculations. From the engineering point of view, pressure and temperature could be the important parameters of nanofluid flow in porous media which should be precisely predicted by theoretical methods. A supplementary method is needed to optimize the CFD approach and make it faster. Artificial intelligence (AI) algorithms have the potential in finding the pattern of simulation data. AI algorithms could learn the CFD results for several specific conditions (e.g. specific positions, times, properties, boundary conditions, etc.), find the general pattern of data, and predict the targeted variables for the other conditions without any further computational expenses. The machine learning of the CFD results was used for the first time by references^15–22^. The researchers used the fuzzy inference system (FIS) with the adaptive network (AN) algorithm for the training process. There are a number of other trainers such as genetic algorithm (GA), ant colony optimization (ACO), neural network (NN), etc. that are available and could be used with integration with FIS. Every AI algorithm that possesses the highest level of accuracy would be called the best intelligent condition. There are several parameters, specifically for each AI algorithm, defining the best intelligence condition. These parameters could be changeable from one CFD case study to another one. The details of the artificial intelligence algorithm and also the effects of the parameters are absent in these studies. Additionally, the extra investigations are required to show the contribution of other trainers like ant colony optimization with the FIS for helping the CFD.

This study tries to do machine learning of the CFD results of a nanofluid flow in a porous media for some specific nanoparticle concentrations. The ant colony optimization-based fuzzy inference system (ACOFIS) is developed, for the first time, for this purpose. After obtaining the best intelligence, the pressure and temperature can be predicted by the ACOFIS for any other values of the nanoparticles concentrations without any other CDF modeling. The computational times of the ACOFIS are presented for such a case as feedback for facilitating the CFD method. The general parameter of input number and the specific one, the pheromone effect related to ant colony optimization, are considered for sensitivity tests of the best intelligence condition.

## Methodology

### Computational fluid dynamics

A cylindrical duct with a length (L) of 1.0 m completely occupied with a porous medium was considered as the geometrical configuration, in which the porous medium is saturated with a circular section with the diameter of 0.01 m (D) and a single phase. The single-phase model with a mixture behaving like a single-phase fluid hypothetically was taken into account in this work. The nanofluid within the single-phase model is considered as a normal fluid, but with improved features owing to the presence of nanoparticles in the system.

The mathematical explanation of single-phase leading equations is represented as follows^23–26^.

Continuity equation:

1 $$\nabla \cdot \left( {\rho_{eff} V} \right) = 0$$

Momentum equation:

2 $$\frac{1}{{\varepsilon^{2} }}\nabla \cdot \left( {\rho_{eff} \vec{V}\vec{V}} \right) = - \nabla p + \frac{1}{\varepsilon }\nabla \left[ {\mu_{eff}^{e} \left( {\nabla \vec{V} + \left( {\nabla \vec{V}} \right)^{T} } \right)} \right] - \frac{{\mu_{eff} }}{K}\vec{V} - \frac{{\varepsilon C_{d} \rho_{eff} }}{\sqrt K }\left| V \right|\vec{V}$$

Energy equation:

3 $$\nabla \cdot \left( {\rho_{eff} VC_{p.eff} T} \right) = \nabla \cdot \left( {\varepsilon k_{eff} \nabla T - \varepsilon \left( {\rho C_{p} } \right)_{eff} \overline{VT} } \right)$$

The parameters of porous media such as porosity ε, inertia coefficient *C*_*d*_ and permeability *K* are reported in references^27–30^.

4 $$K = 0.00073d_{pore}^{2} \left( {1 - \varepsilon } \right)^{ - 0.224} \left( {\frac{{d_{cell} }}{{d_{pore} }}} \right)^{ - 1.11}$$

5 $$C_{d} = 0.00212\left( {1 - \varepsilon } \right)^{ - 0.132} \left( {\frac{{d_{cell} }}{{d_{pore} }}} \right)^{ - 1.63}$$

6 $$d_{pore} = 0.0254\left( m \right)/10\left( {PPI} \right)$$

7 $$d_{cell} = 1.18d_{pore} \sqrt {\frac{1 - \varepsilon }{{3\pi }}} \left( {\frac{1}{{1 - e^{{\frac{{ - \left( {1 - \varepsilon } \right)}}{0.04}}} }}} \right)$$

Effective thermal conductivity is influenced by nanofluid thermal conductivity and the copper porous conductivity and is given as follows^27,30^:

8 $$k_{eff} = \left( {1 - \varepsilon } \right)k_{porous} + \varepsilon k_{nf}$$

The effective thermophysical properties of the nanofluid are collected in Table 1. Considering the effect of Brownian phenomenon, Chon et al.^31^ correlation is adopted for the calculation of thermal conductivity, while the correlation suggested by Masoumi et al.^32^ is employed for estimating the fluid viscosity. So, for using such temperature-dependent correlations of conductivity and viscosity, User Defined Function (UDF) codes have been developed and added to the ANSYS-FLUENT CFD codes.

**Table 1:** Al_2_O_3_/water properties.

Properties	Equation
Density^23^	$\rho_{eff} = \left( {1 - \varphi } \right)\rho_{f} + \varphi \rho_{p}$
Heat capacity^23^	$c_{p,eff} = \frac{{\left( {1 - \varphi } \right)(\rho c_{p} )_{f} + \varphi (\rho c_{p} )_{p} }}{{\left( {1 - \varphi } \right)\rho_{f} + \varphi \rho_{p} }}$
Viscosity^32^	$\mu_{eff} = \left( {1 + \frac{{\rho_{np} v_{B} d_{p}^{2} }}{72C\delta }} \right)\mu_{f}$ $v_{B} = \frac{1}{{d_{p} }}\sqrt[3]{{\frac{{18K_{B} ~T}}{{\pi \rho _{{np}} d_{p} }}}}$ $\delta = \sqrt[3]{{\frac{{\pi d_{p} }}{6\phi }}}$
Thermal conductivity^31^	$k_{nf} /k_{bf} = 1 + 64.7\left( \phi \right)^{0.7460} \left( {\frac{{d_{bf} }}{{d_{p} }}} \right)^{0.3690} \left( {\frac{{k_{bf} }}{{k_{p} }}} \right)^{0.7476} Pr^{0.9955} Re_{np}^{1.2321}$ $Re_{np} = \frac{{\rho_{bf} K_{B} T}}{{3\pi \mu_{bf}^{2} \lambda }}$

The $k - \varepsilon$ turbulence model to calculate the turbulent eddy viscosity, its energy dissipation rate $\left( \varepsilon \right)$, and the turbulent kinetic energy $\left( k \right)$ are based on the literature^23,33,34^:

9 $$\nabla \cdot (\rho_{eff} kV) = \nabla \cdot \left[ {\left( {\frac{{\mu_{t} }}{{\sigma_{k} }}} \right)\nabla (k)} \right] + G_{k} - \rho_{eff} \varepsilon$$

10 $$\nabla \cdot (\rho_{eff} \varepsilon V) = \nabla \cdot \left[ {\frac{{\mu_{t} }}{{\sigma_{\varepsilon } }}\nabla \varepsilon } \right] + \frac{\varepsilon }{k}(C_{1\varepsilon } G_{k} - C_{2\varepsilon } \rho_{eff} \varepsilon )$$

11 $$G_{k} = \mu_{t} (\nabla V + (\nabla V)^{T} ), \, \mu_{t} = \rho_{eff} C_{\mu } \frac{{k^{2} }}{\varepsilon }$$

$$C_{\mu } = 0.09,\sigma_{k} = 1.00,\sigma_{\varepsilon } = 1.30,C_{1\varepsilon } = 1.44,C_{2\varepsilon } = 1.92$$

#### CFD grid test and validation

The mesh dependency test has been carried out for two different grid arrangements (i.e. 107,400 nodes, and 161,100 nodes). According to the CFD results and testing both mesh sizes, the relative temperature and velocity differences were less than 0.05%. So, the first mesh size was selected for the calculations due to less computational expenses and shorter solution time.

For verifying the CFD outputs, calculated Nusselt numbers (Nu) are compared with those measured data of Fotukian and Esfahany^35^ investigations. According to Table 2, the CFD results for the simple tube (without porous) are the same as those of the experiment. Inserting the porous material inside the pipe, the Nu becomes threefold.

**Table 2:** Nusselt number calculation (CFD and experiment comparison).

Study	Fluid	Tube type	Re	Nu
Fotukian and Esfahany^35^	0.054% Al_2_O_3_/water	Simple tube	9,950	82.95
	0.14% Al_2_O_3_/water	Simple tube	7,084.23	67.07
	0.14% Al_2_O_3_/water	Simple tube	10,799.1	86.13
Present study	0.3% Al_2_O_3_/water	Simple tube	10,000	85.86
	2% Al_2_O_3_/water	Simple tube	10,000	99.13
	2% Al_2_O_3_/water	Porous tube	10,000	306.27

### Ant colony optimization (ACO)

Ant System (AS) is the first ant colony optimization method aimed at finding the shortest routes for linking some cities^36^. The same concept is used in development of ACO algorithm^37^. The colony’s experience is reflected by the pheromone factor, however, the heuristic factor deals with the interest in choosing a component based on an objective function^38^. These parameters are weighted through $\alpha$ and $\beta$ as^38,39^:

12 $$P_{ij}^{k} = \frac{{\left[ {\tau_{ij} } \right]^{\alpha } \left[ {\eta_{ij} } \right]^{\beta } }}{{\mathop \sum \nolimits_{l} \in N_{i}^{k} \left[ {\tau_{il} } \right]^{\alpha } \left[ {\eta_{il} } \right]^{\beta } }},\,\,\,\, if\,\,j \in N_{i}^{k}$$

Pheromone trails are updated when all of the ants create their tours^38,39^:

13 $$\tau_{ij} \leftarrow \left( {1 - \rho } \right)\tau_{ij} , \forall \left( {i,j} \right) \in L$$

Furthermore, by leaving pheromone over the arcs crossed by the ants in their pathway and by the superior the tour, the higher the quantity of pheromone will be received for the arcs^38,39^:

14 $$\begin{gathered} \tau_{ij} \leftarrow \tau_{ij} + \mathop \sum \limits_{k = 1}^{n} \Delta \tau_{ij}^{k} , \forall \left( {i,j} \right) \in L \hfill \\ \Delta \tau_{ij}^{k} = \left\{ {\begin{array}{*{20}c} {\frac{1}{{C^{k} }},} & { if arc \left( {i,j} \right)belongs to T^{k} ;} \\ {0,} & {otherwise;} \\ \end{array} } \right. \hfill \\ \end{gathered}$$

The first enhancement of initial ant system was known as the elitist approach for AS. It deals with providing robust additional reinforcement for the arcs related to the best tour found since starting the method^38–40^:

15 $$\begin{gathered} \tau_{ij} \leftarrow \tau_{ij} + \mathop \sum \limits_{k = 1}^{n} \Delta \tau_{ij}^{k} + e\Delta \tau_{ij}^{bs} , \forall \left( {i,j} \right) \in L \hfill \\ \Delta \tau_{ij}^{bs} = \left\{ {\begin{array}{*{20}c} {\frac{1}{{C^{bs} }},} & { if arc \left( {i,j} \right)belongs to T^{bs} ;} \\ {0,} & {otherwise;} \\ \end{array} } \right. \hfill \\ \end{gathered}$$

The rank-based version is another enhancement over ant system. Each ant deposits some pheromone decreasing by its rank. Moreover, the largest quantity of pheromone is always deposited by the best-ant-so-far during every iteration^38–40^.

16 $$\tau_{ij} \leftarrow \tau_{ij} + \mathop \sum \limits_{r = 1}^{w - 1} \left( {w - r} \right)\Delta \tau_{ij}^{r} + \Delta \tau_{ij}^{bs}$$

### Fuzzy inference system (FIS)

The FIS is utilized in the fields of optimization, control, classification and prediction in engineering sciences. Here, if–then rules are employed to design the FIS structure^41^. In this rule, the signals are multiplied based on the AND rule as the node function. For case, the i^th^ rule function can be written as^41^:

17 $$w_{i} = \emptyset \left( X \right) \delta \left( Y \right)\sigma \left( {NVF} \right)$$

The firing strength value is determined for each rule, as following^16,41^:

18 $$\overline{w}_{i} = \frac{{w_{i} }}{{\sum \left( {w_{i} } \right)}}$$

where $\overline{w}_{i}$ is determined as normalized firing strengths. If–then rule developed by Takagi and Sugeno^41^.

Consequently, the node function is:

19 $$\overline{w}_{i} f_{i} = \overline{w}_{i} \left( {p_{i} X + q_{i} Y + r_{i} NVF + s_{i} } \right)$$

Detailed descriptions of FIS can be found in our previous publications^16,42–46^.

## Results and discussions

This study tries to develop a new artificial intelligence algorithm using the ant colony optimization, as a trainer, in a combination with the fuzzy inference system. The hybrid algorithm is then called ACOFIS. The ability of the developed algorithm is tested in the pressure and the temperature predictions of the Al_2_O_3_/H_2_O NF in a porous tubular duct. Machine learning (ML) of the CFD results is employed in order to recognize the pattern of the flow characteristics. The pressure and temperature of the nanofluid on the cross-section plate at 0.3 m from the inlet are considered for such predictions in this study. Each trainer has its own parameters playing crucial roles in the accurate predictions of the data patterns extracted from the CFD computations. These parameters could be also changeable from one CFD case study to another. The pheromone effect is one of the important parameters of ant colony optimization. The effect of such parameters in the accurate prediction of CFD results is investigated in this study. The accuracy of the ACOFIS is checked with the CFD results and the predictions of the widely used artificial intelligence of ANFIS.

**Figure 1:**
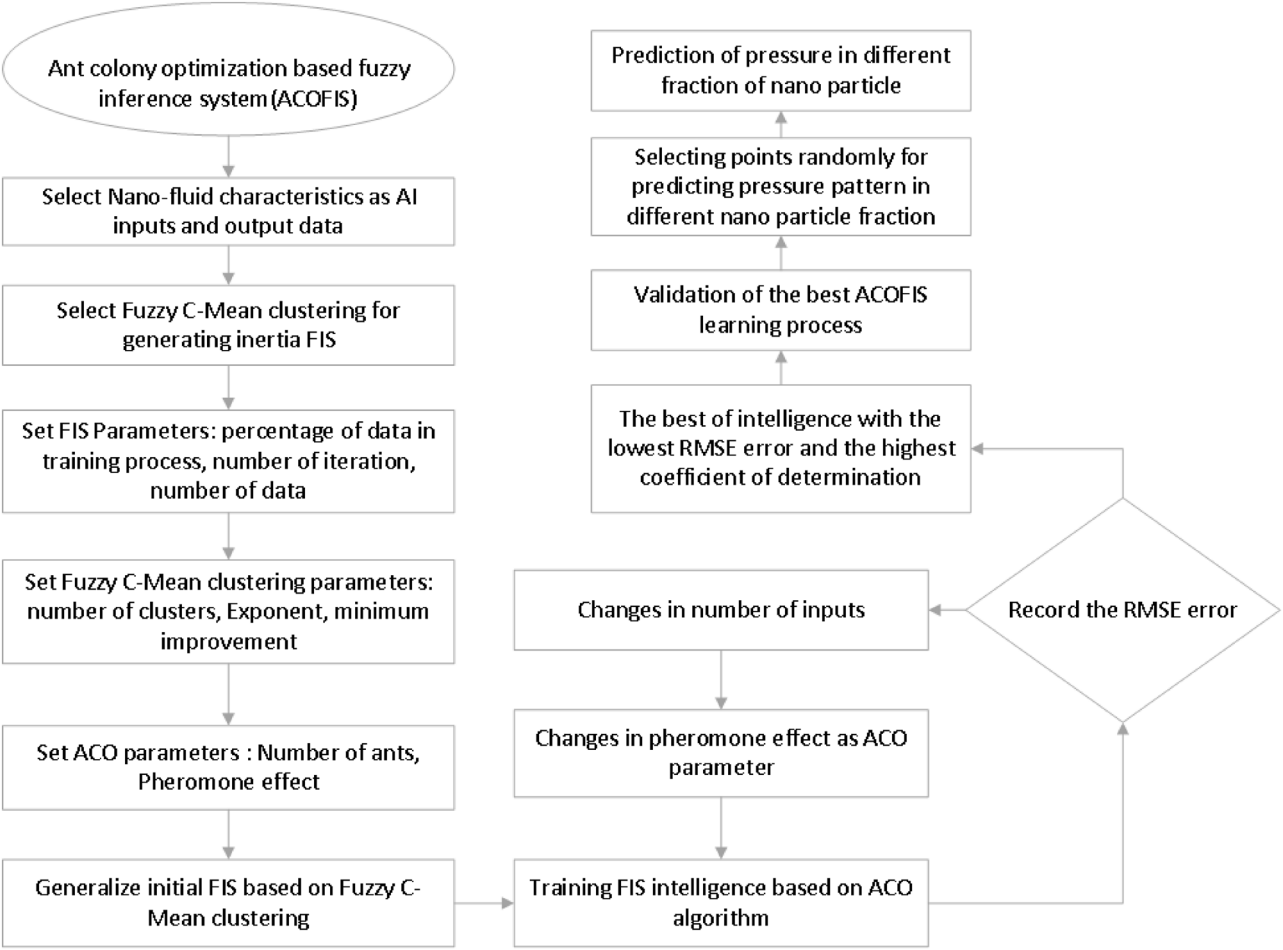
Schematics of combination of ant colony algorithm with fuzzy system.

Figure 1 shows flowchart of using ACO as trainer in fuzzy inference system for learning the CFD results. As seen, the predicted pressures and temperatures are compared with the CFD calculations. The intelligence of model is checked by the root mean square error (RMSE) and the regression number (R). RMSE and the R values are recorded for different input numbers and pheromone effects. The lowest RMSE and the highest R means that the ACOFIS has achieved the best intelligence. At the best intelligence, for further validation, several nodes are selected randomly. The ACOFIS predicts nanofluid pressures and temperatures on the selected nodes for any values of the nanoparticles fraction.

**Figure 2:**
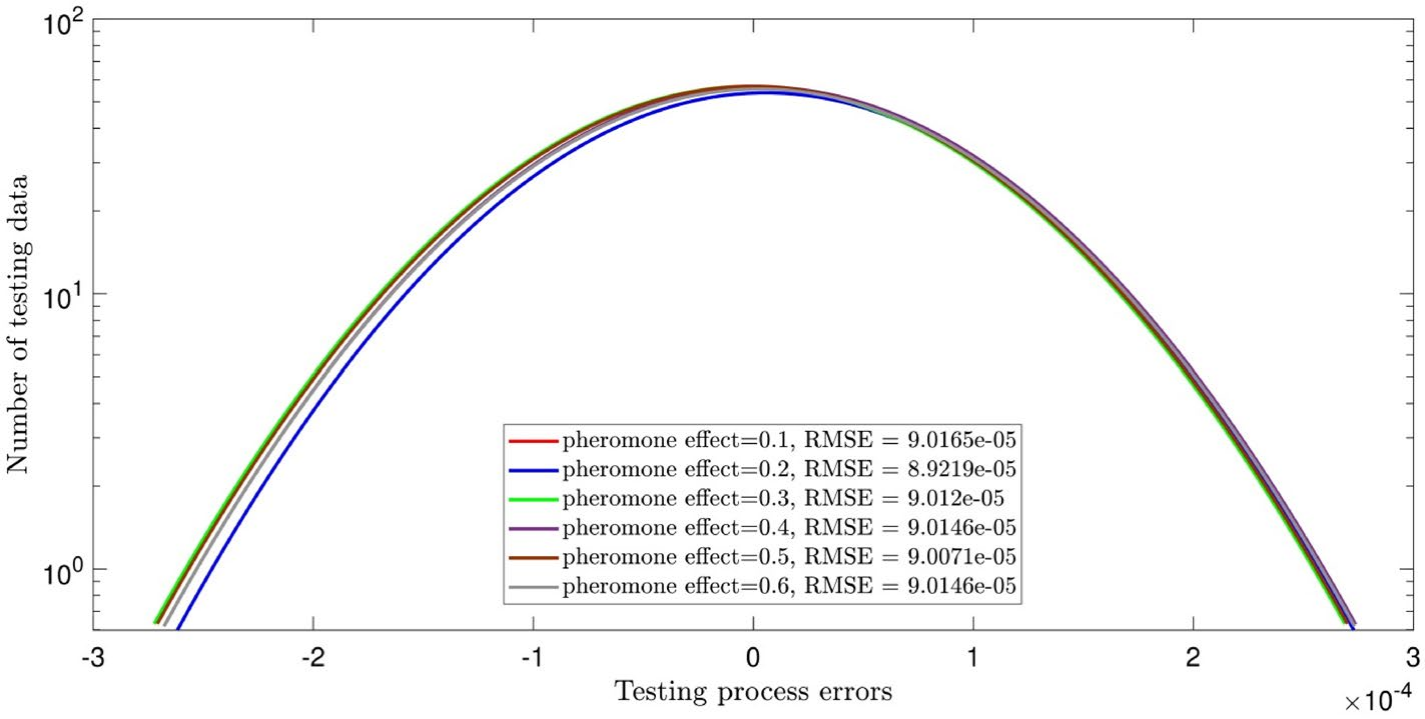
ACOFIS learning processes using two inputs and diversity of pheromone effect.

**Figure 3:**
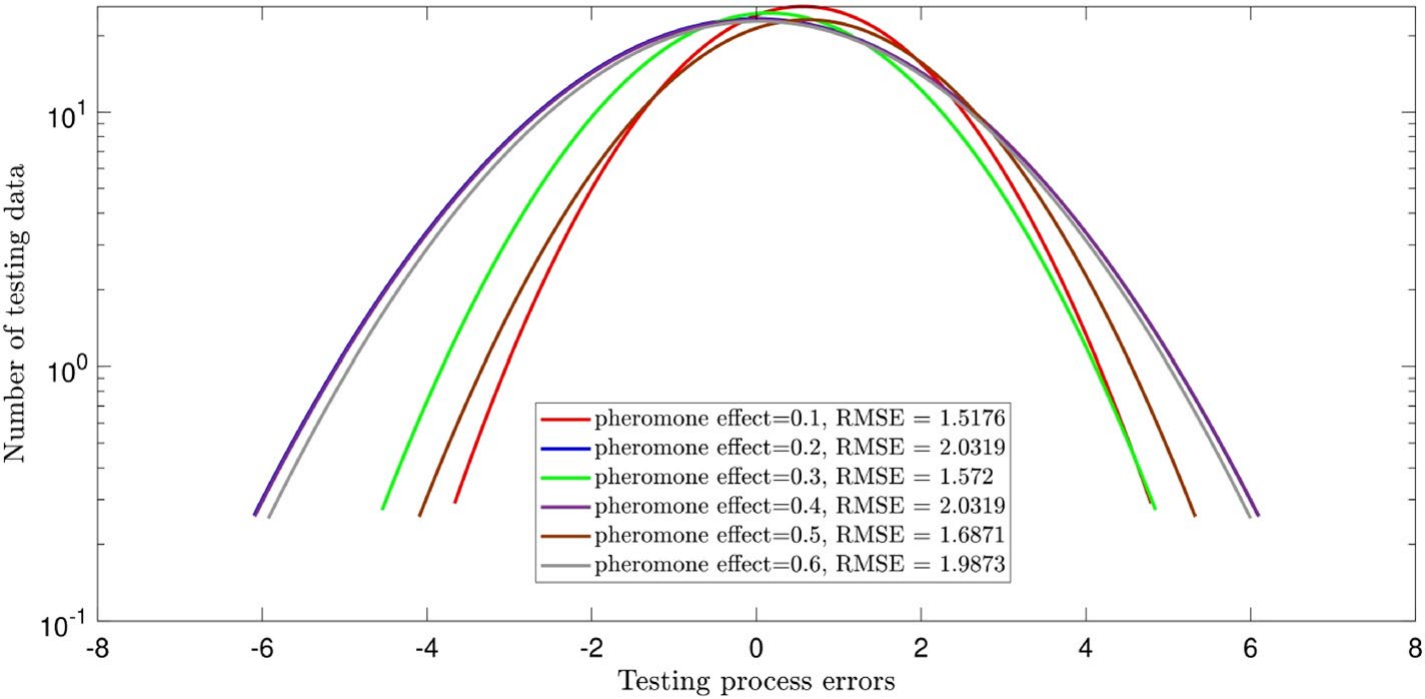
ACOFIS learning processes using three inputs and diversity of pheromone effect.

**Figure 4:**
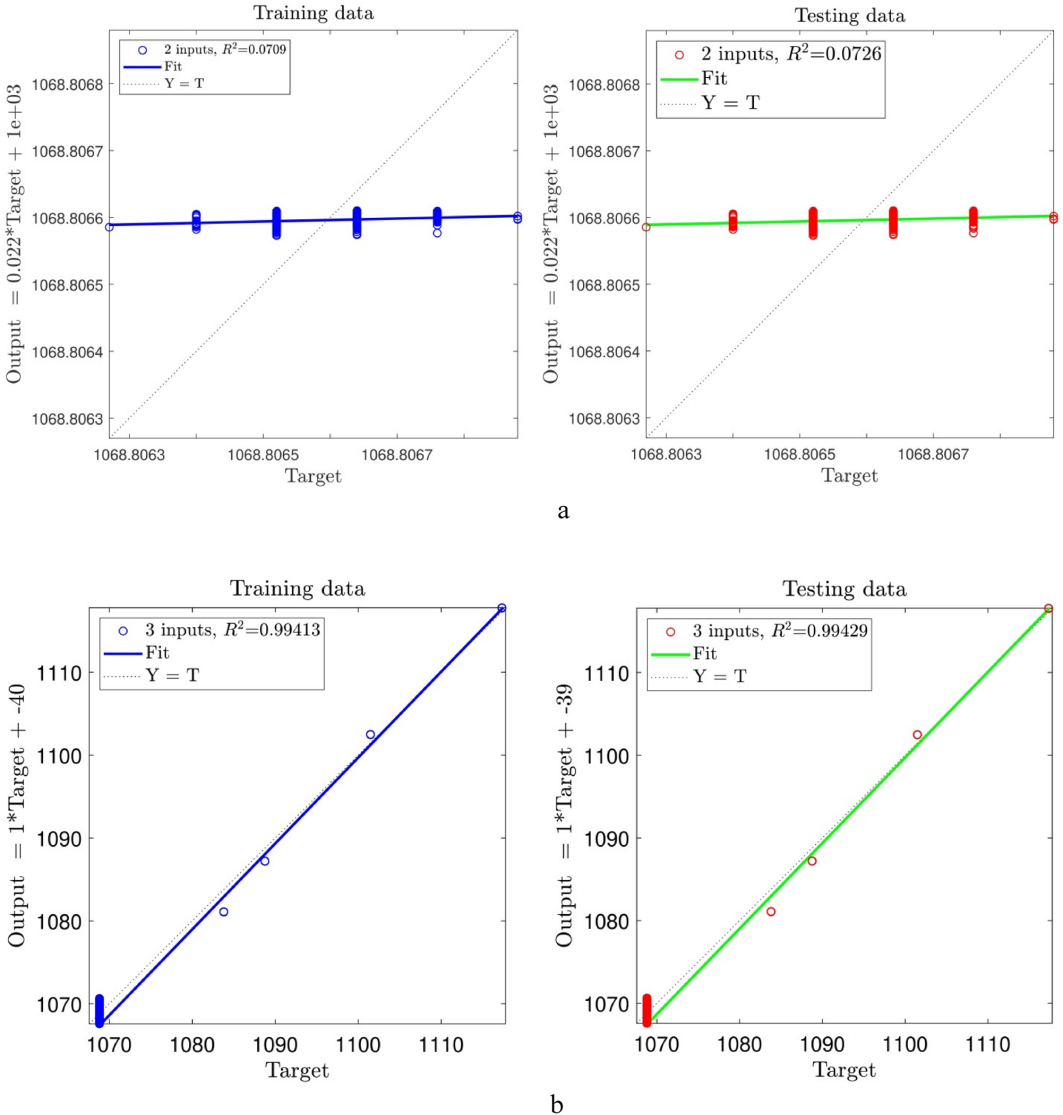
(**a**) Regression plot of the best of ACOFIS intelligence using two inputs in learning process. (**b**) Regression plot of the best of ACOFIS intelligence using three inputs in learning process.

Herein, the details of the tuning analysis of the ACOFIS are given just for the pressure prediction. Figure 2 shows the histogram error distribution graph for two inputs and different values of the pheromone effect. No change is seen by increasing the pheromone effect. Indeed, the error is distributed between ± 3, and the RMSE is around 9 × 10^–5^ for all values of the pheromone effect. Adding the nanoparticles volume fraction as the third input, the RMSE increases to the higher values (i.e. between 1.5 and 2) as illustrated in Fig. 3. According to Fig. 3, the error distribution and the RMSE are sensitive to the pheromone effect. The least RMSE (i.e. 1.5) is for the pheromone effect of 0.1 and the errors are distributed between ± 4. Although the RMSE value of 3 inputs is higher than that of 2 inputs, the ACOFIS reaches the best intelligence for the case when 3 inputs have been considered. This is shown in Fig. 4; the increase of input number from 2 to 3 leads to the increase of regression number from 0.07 to 0.99. Therefore, the RMSE only is not enough for intelligence determination. The RMSE increment by the number of inputs could be justified by the increase of data from 547 for 2 inputs to 2685 for 3 inputs. In fact, learning more data leads to more RMSE.

A similar analysis could be done for the best condition in prediction of temperature. A summary of this analysis and its results are given in Table 3. Unlike the pressure, the pheromone effect is equal to 0.4 at the best intelligence in prediction of temperature. This means that AI algorithms should be tuned for the prediction of each type of parameter.

**Table 3:** ACOFIS setup for predicting pressure and temperature in the same setup.

	ACOFIS for predicting pressure	ACOFIS for predicting temperature
CFD case study	Nanofluid turbulent flow in heated porous pipe	Nanofluid turbulent flow in heated porous pipe
AI method	Combination of ACO with FIS	Combination of ACO with FIS
Material of case study	Nanofluid (Al_2_O_3_)	Nanofluid (Al_2_O_3_)
Number of input in the best intelligence	3	3
Pheromone effect in the best intelligence (ACO parameter)	0.1	0.4
Changes in number of inputs was evaluated(FIS parameter)	2,3	3
Changes in pheromone effect was evaluated(ACO parameter)	0.1, 0.2, 0.3, 0.4, 0.5, 0.6	0.1, 0.2, 0.3, 0.4, 0.5, 0.6
The highest of correlation coefficient in testing process with 100% of data	0.994	0.968
P(%) percentage of used data in training process	77%	77%
Number of data	2 inputs (537) and 3 inputs (2685)	3 inputs (2685)
Number of iteration	115	115
Type of data clustering	FCM clustering	FCM clustering
Type of membership function	Guassmf	Guassmf
Number of MFs for each input	16	16
Number of rules (which is for hidden layer of FIS)	16	16
Number of membership functions (MFs) for output	16	16
ACOFIS input1	x-direction	x-direction
ACOFIS input2	y-direction	y-direction
ACOFIS input3	Nano particle Fraction = 0.5,0.8,1,1.5,2%	Nano particle Fraction = 0.5,0.8,1,1.5,2%
ACOFIS output	Pressure	Temperature

**Figure 5:**
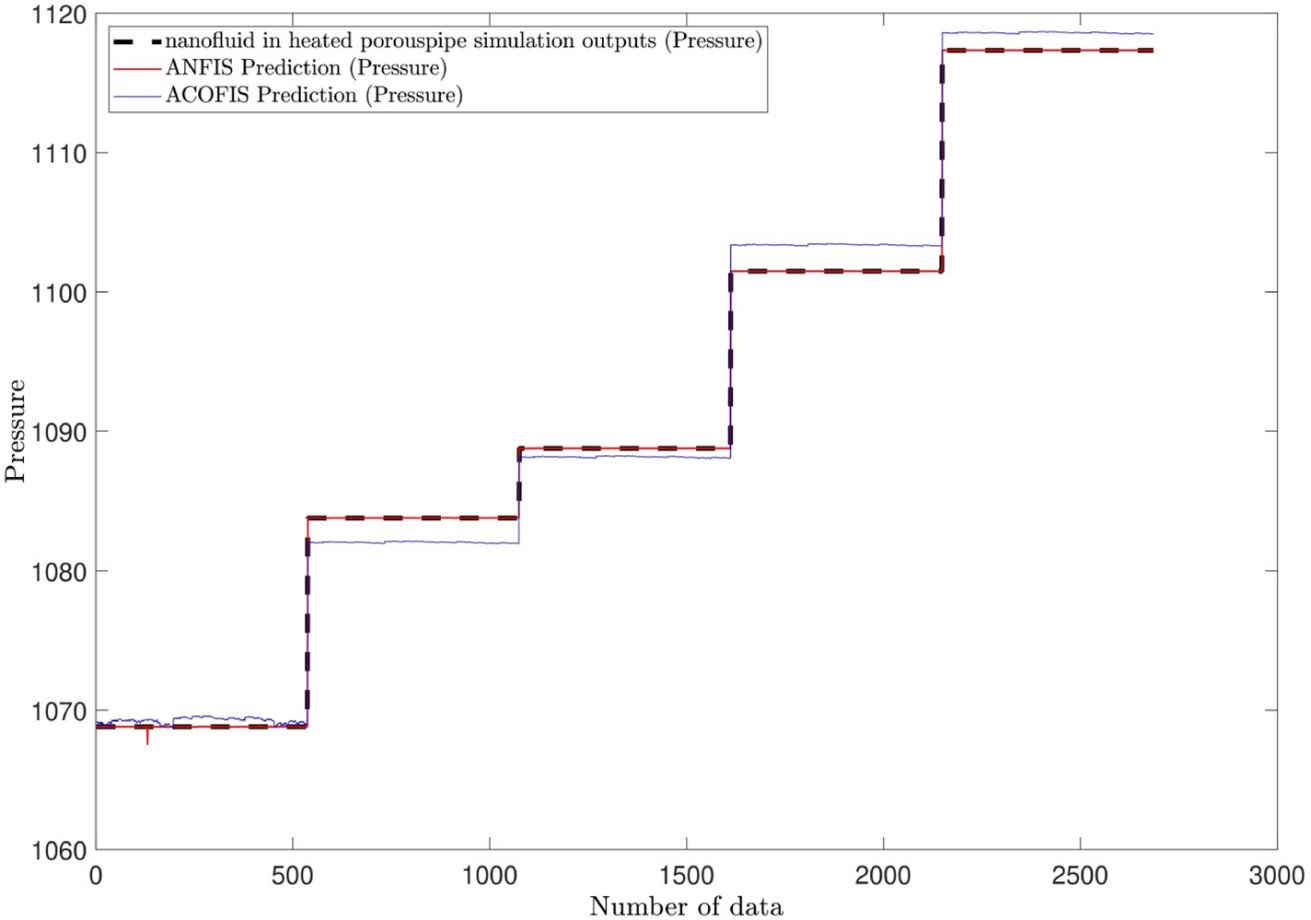
Comparison of pressure prediction with different AI methods.

For more validation, the predictions of the newly developed AI algorithm of ACOFIS are compared with the widely used algorithm of ANFIS. Table 4 explains this comparison. For similar iteration number, input number and clustering type, both methods achieve the highest values (around 1) of the correlation coefficient (R) and the coefficient of determination (R^2^) in their predictions. The RMSE of ACOFIS (~ 1.3) is a little more than that of ANFIS (~ 0.03). This is shown in Fig. 5 where a comparison has been made between the ACOFIS and the ANFIS predictions of pressure for all learned data. The CFD results have been also shown as a benchmark. The ACOFIS predictions are close to the ANFIS and CFD results with a little deviation.

**Table 4:** Detail of ACOFIS and ANFIS setup and learning times.

Method	ACOFIS method	ANFIS method
Number of inputs	3	3
Percentage of data in training process	77	77
Number of iterations	115	115
Clustering Type	Fuzzy C-mean Clustering	Fuzzy C-mean Clustering
Exponent as FCM clustering parameter	2	2
Minimum Improvement as FCM clustering parameter	1.00E-05	1.00E-05
Correlation coefficient (R) in training process	0.997867326	0.99999993
Coefficient of determination (R^2^) in training process	0.995739201	0.99999986
RMSE error in testing process	1.331970484	0.025145357
Correlation coefficient (R) in testing process	0.997857536	0.999998829
Coefficient of determination (R^2^) in testing process	0.995719663	0.999997657
Learning process time (s)	198.0029725	212.5959348
Prediction process time (s)	0.1300083	0.519412

The learning time and prediction time of the ANFIS (212.6 s and 0.5 s respectively) are a little bit more than those of the ACOFIS (198 s and 0.1 s respectively). Although the total time difference is not that much (less than 15 s), this could be significant in actual large-scale cases. Totally, the summation of the learning and the prediction times take just a few minutes (less than 4 min). This means the AI algorithms could be so fast and as a result, need a little computational requirement in comparison with challenging and expensive CFD computations.

**Figure 6:**
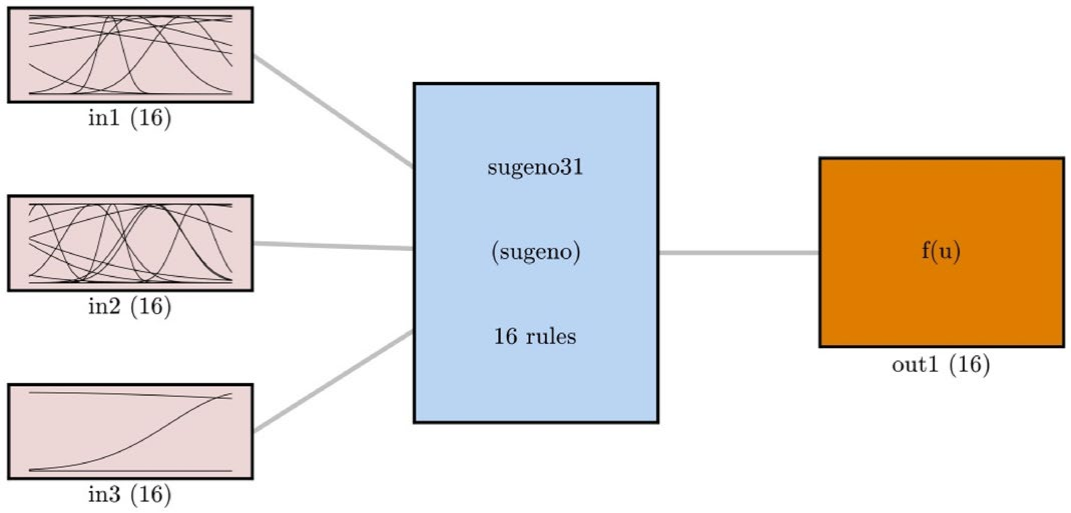
FIS structure in the best results.

Figure 6 describes the number of membership functions (MFs) for each input, the number of rules, and the number of MFs for the output. All these numbers are equal to 16.

**Figure 7:**
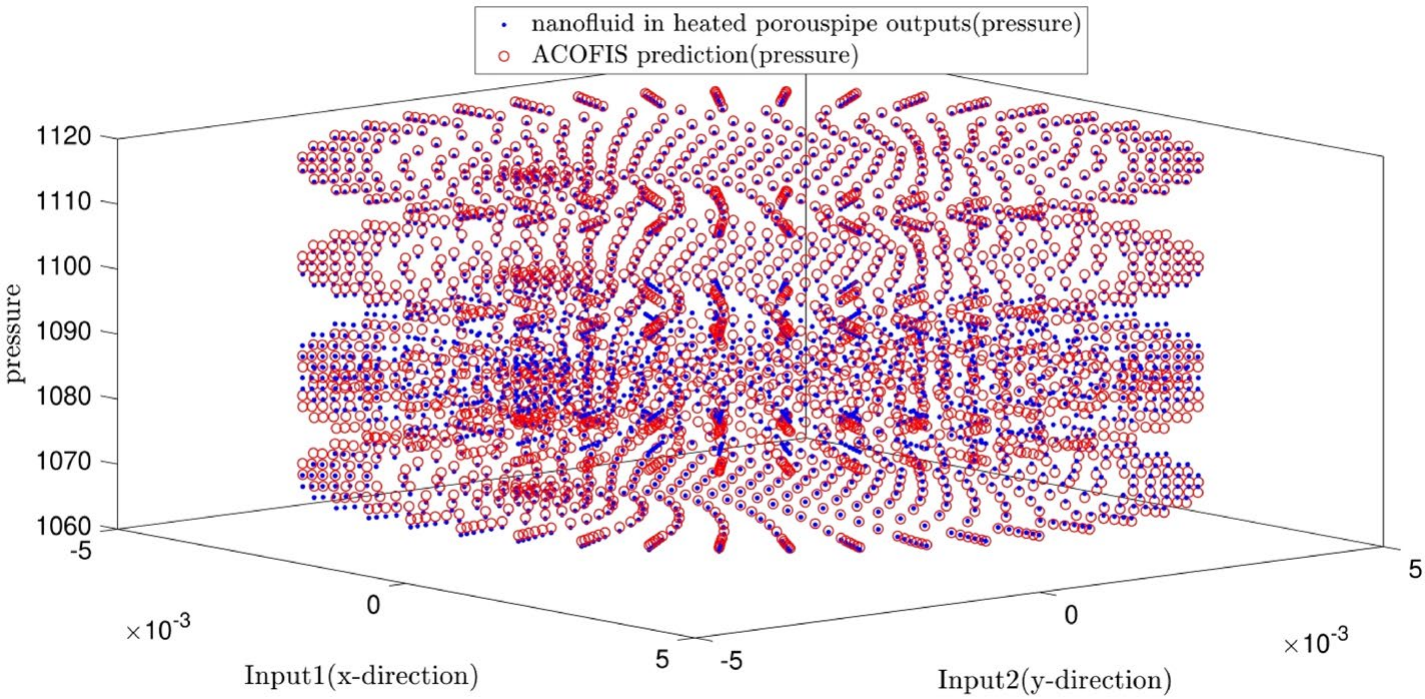
ACOFIS validation with comparison prediction data and CFD data.

**Figure 8:**
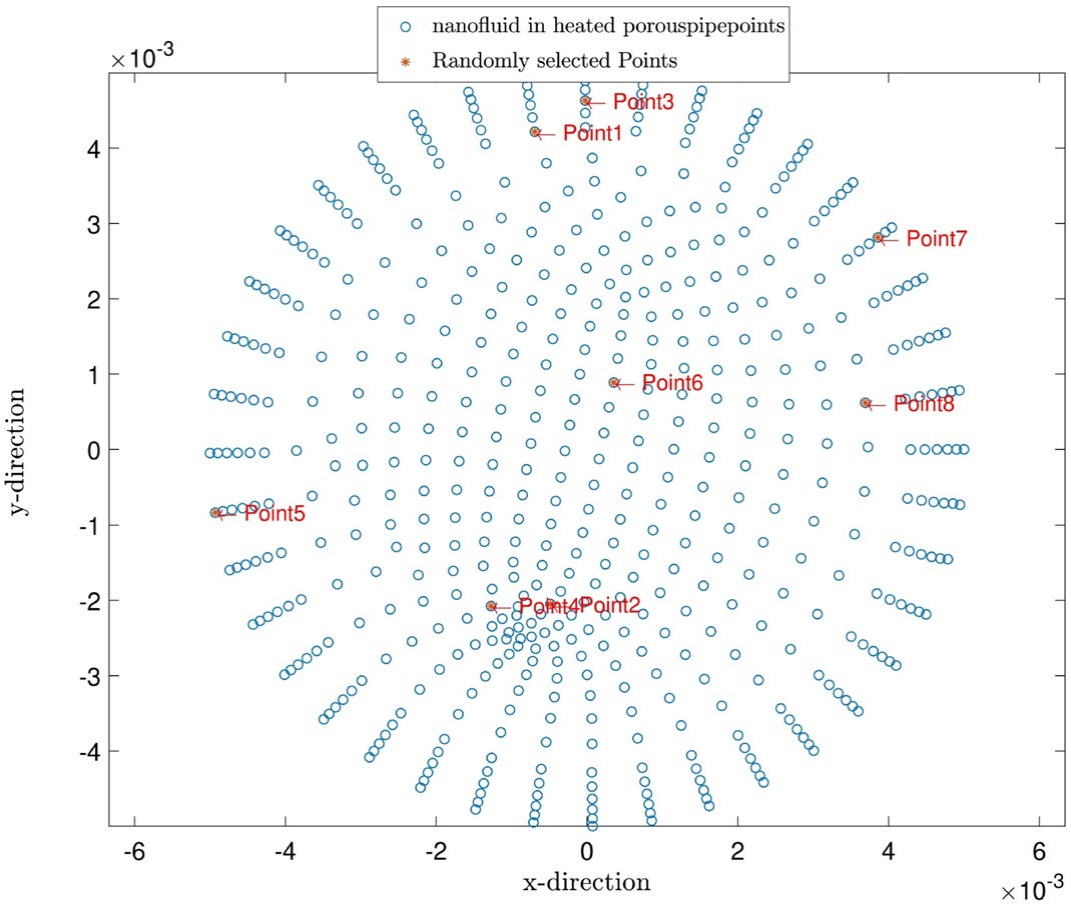
Random points for pattern recognition of pressure in different nanoparticle fractions.

**Figure 9:**
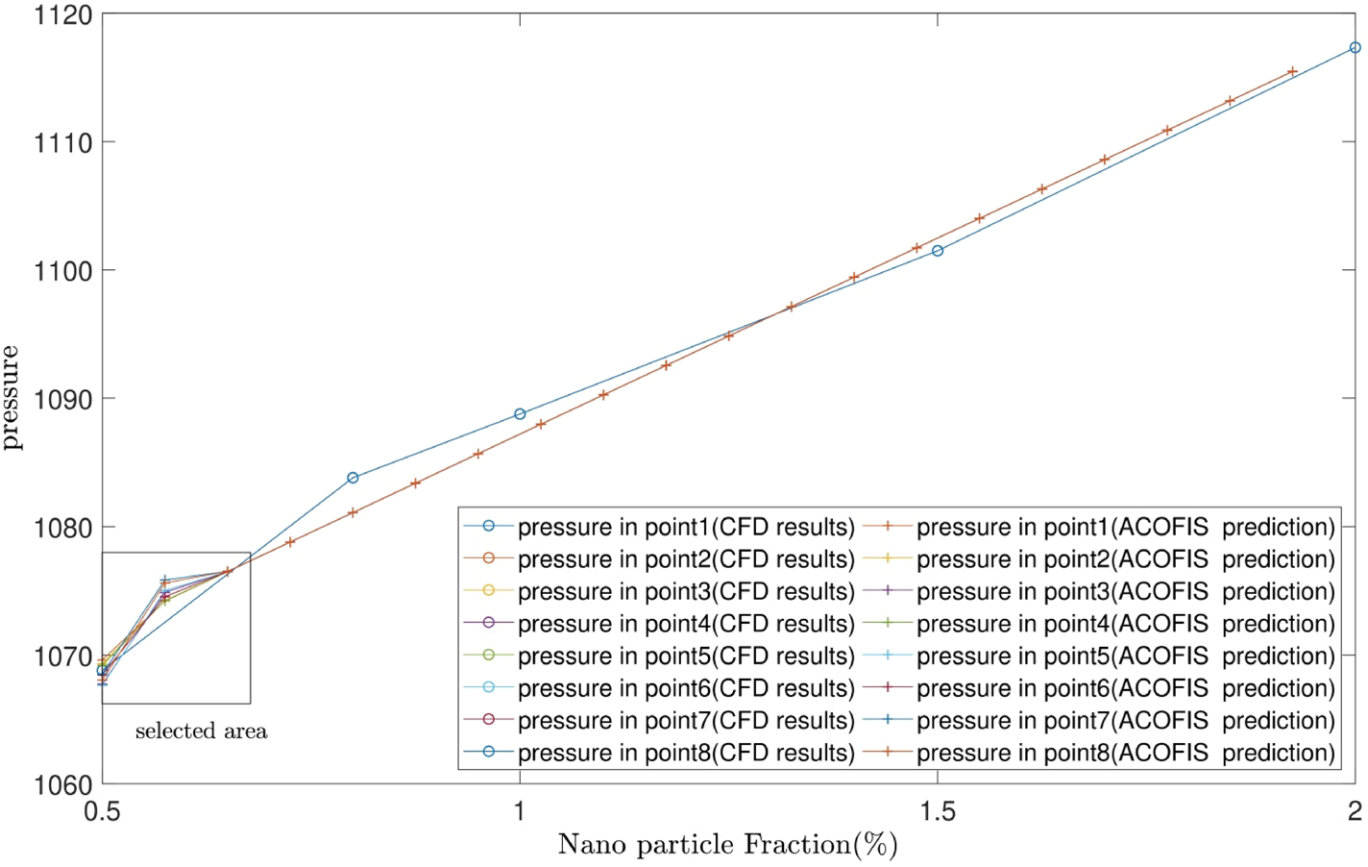
Pattern prediction of pressure in different Nano particle fractions.

**Figure 10:**
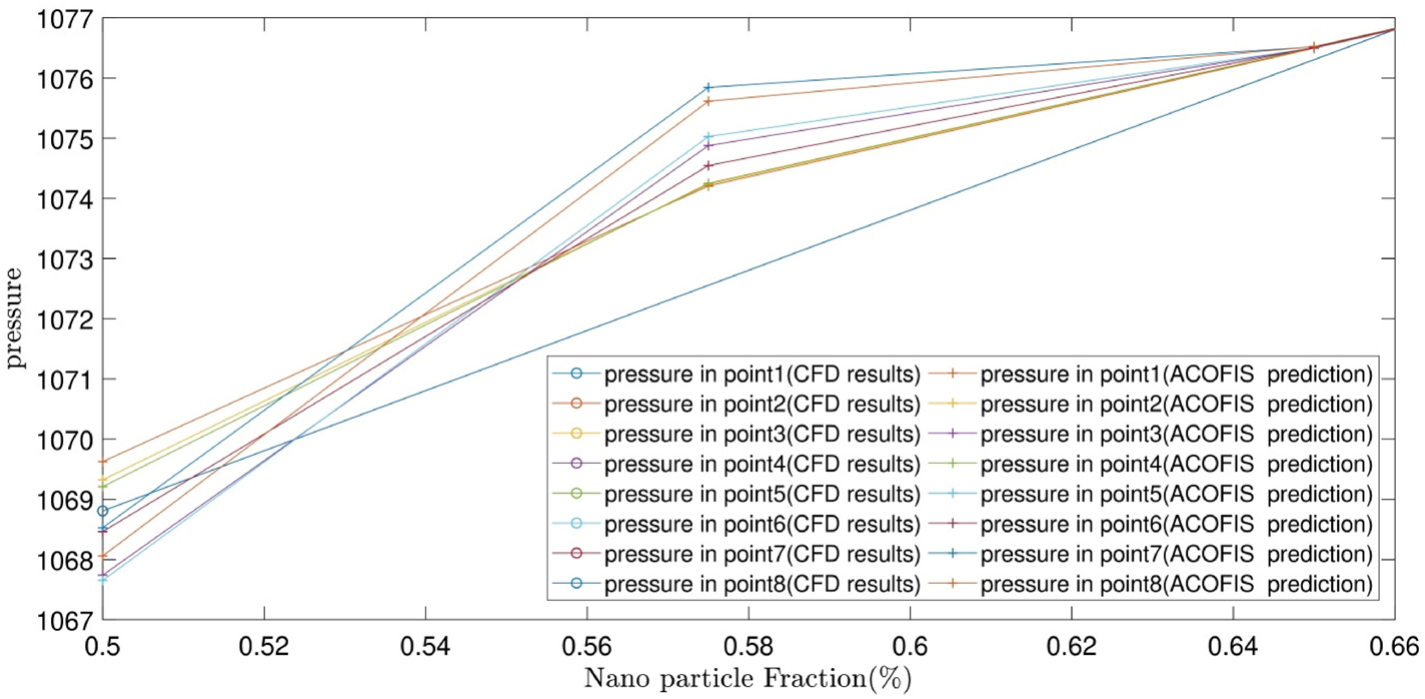
Selected area in Fig. 9.

Figure 7 illustrates the predicted pressure of the nanofluid at different nanoparticles volume fractions by both CFD and ACOFIS models. The red points are the pressures of the nodes predicted by the ACOFIS, while the blue ones are the data simulated by the CFD. The ACOFIS predictions are the same as the CFD. Both methods show the increase of the pressure by nanoparticles volume fraction increment. 8 nodes on the cross-section plate are randomly selected (as shown in Fig. 8). Figure 8 illustrates the pressure prediction of the selected nodes for some other nanoparticle volume fractions. According to Figs. 9 and 10, there is good compatibility between the predicted pressures of both methods specifically for higher nanoparticle fractions (i.e. more than 0.66%).

**Figure 11:**
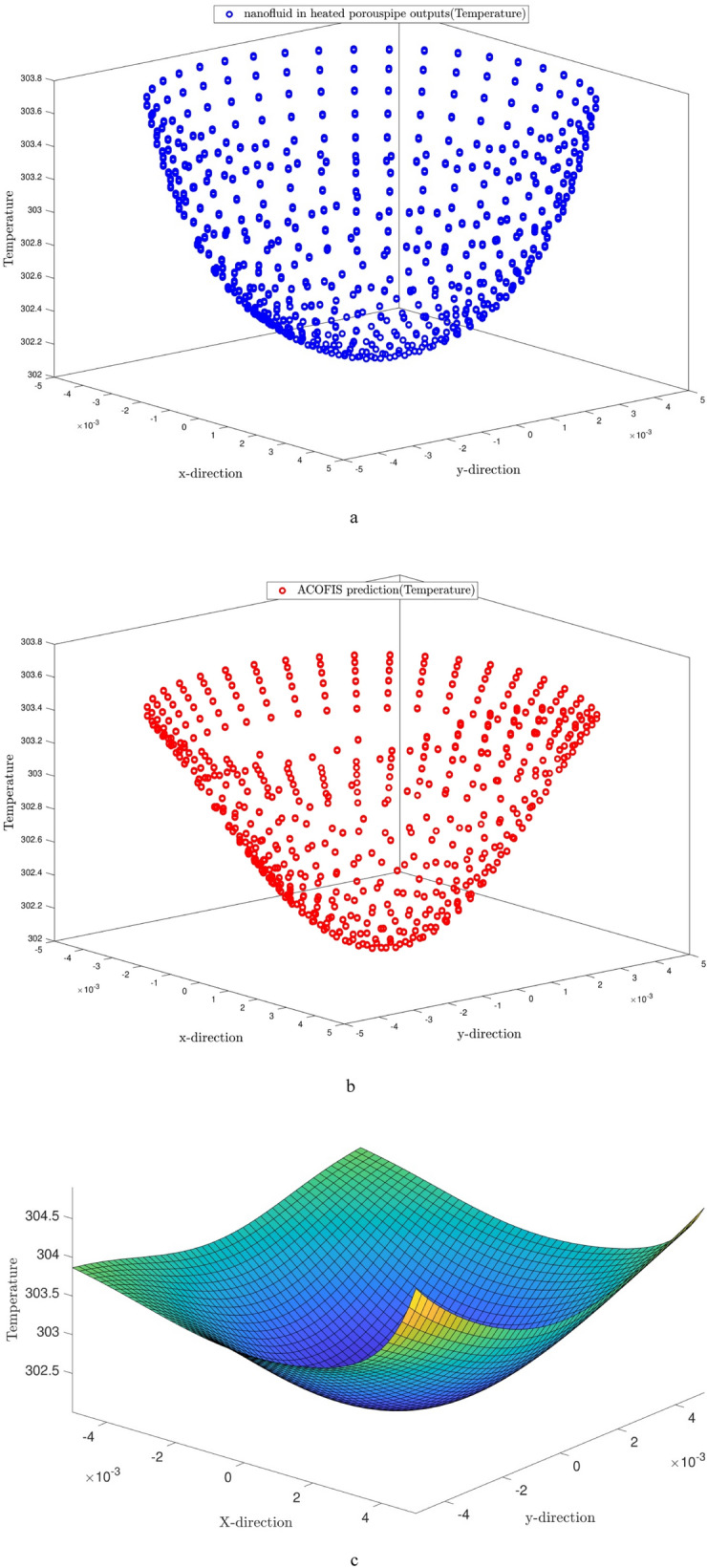
(**a**) CFD simulation results. (**b**) ACOFIS temperature prediction. (**c**) ACOFIS prediction surface for predicting temperature in different points.

**Figure 12:**
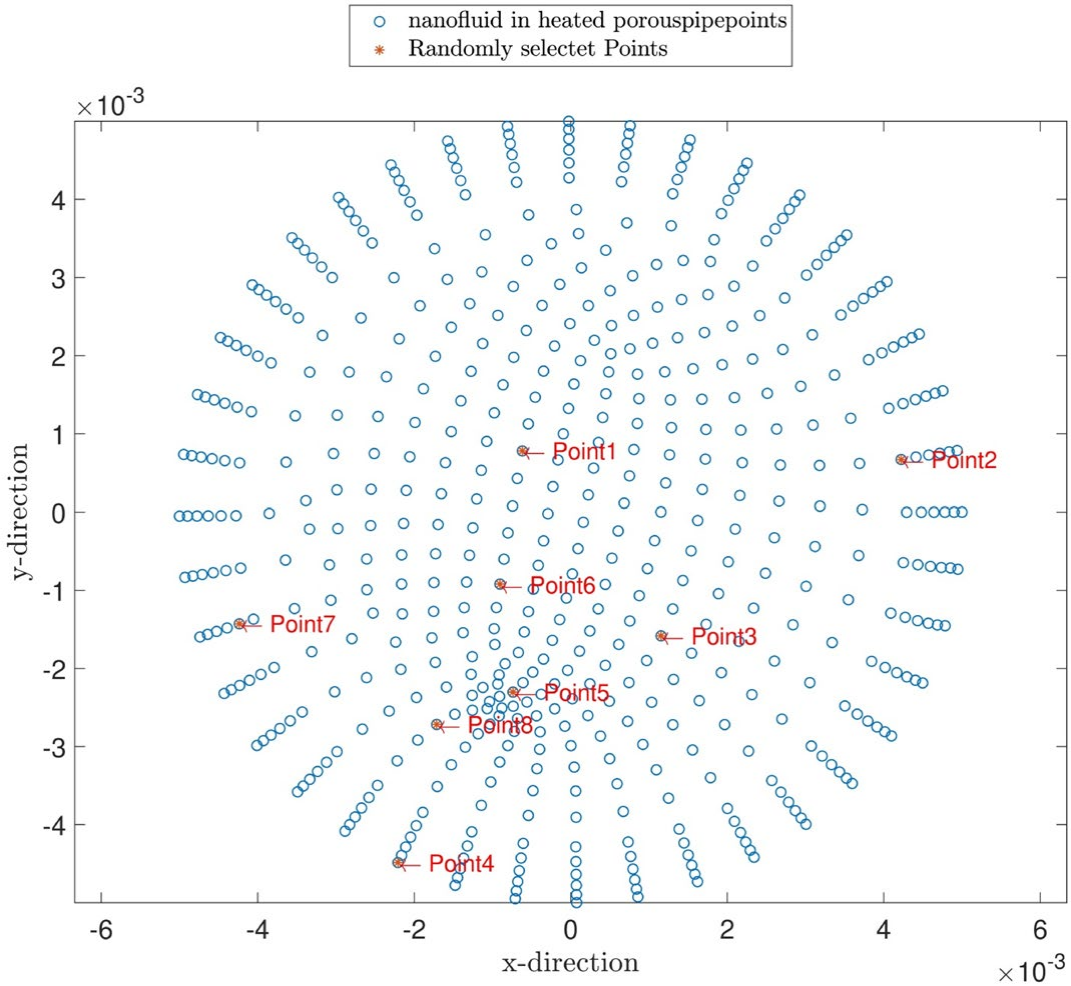
Random selected points for prediction of temperature in different nanoparticle fractions.

**Figure 13:**
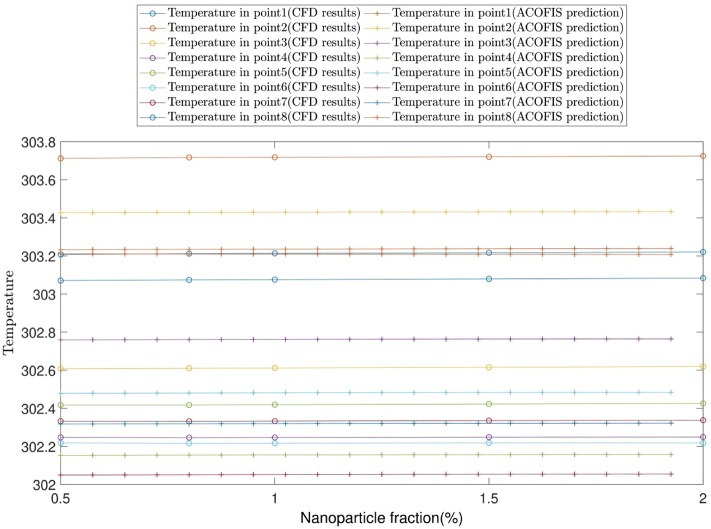
Pattern of predicted temperature in different nanoparticle fractions.

All the above-mentioned procedures are repeated for the temperature predictions of the nanofluid at different volume fractions. Figure 11 illustrates the temperature contour of the nanofluid. According to Fig. 11a,b, the temperature predictions by the ACOFIS are compatible with the CFD. A curved surface is fitted to temperature predictions of the ACOFIS. This surface could give the temperature in any random location on the domain. For example, several nodes randomly selected from the domain, as shown in Fig. 12. The pattern of changing temperature in selected points by the nanoparticle fraction has been predicted and depicted in Fig. 13. This means there is no need for more CFD calculations for new nanoparticle fractions. The fast ACOFIS calculation could be replaced with the time-consuming method of CFD.

## Conclusions

Although the CFD methods have a progressive trend in the precise prediction of the physical phenomena, there is not enough evidence for optimization of the CFD approach complexities. They might take more CPU time or require more computational hardware requirements especially in complex problems. So, another method is required for the CFD simplification. The machine learning (ML) of the artificial intelligence (AI) algorithms has shown the significant roles in finding the general patterns of the CFD results. In this case, the adaptive network (AN), as a data trainer, combining with the fuzzy inference system (FIS) has been commonly used. Each trainer has its own specific tuning parameters, and the accurate prediction of the AI algorithms are so dependent on the proper selection of these parameters. But there are no investigations available for the efficiency comparison between the AN and the other trainers.

So, this study was aimed to develop the combination of the ant colony optimization (ACO) algorithm with the fuzzy inference system (ACOFIS) for learning the CFD results. This integration of the ACOFIS and the CFD was used for pressure and temperature predictions of Al_2_O_3_/water nanofluid flow in a heated porous pipe. Firstly, the case was simulated by the ANSYS-FLUENT CFD package. Considering the effect of the Brownian motion, User Defined Function (UDF) codes have been developed for effective conductivity and viscosity. The UDF codes were added to the ANSYS-FLUENT commercial CFD codes. Then the ACOFIS learned the CFD results including the pressure and the temperature of the nanofluid on the cross-section plate at z equal to 0.3 m. The intelligence of the ACOFIS was investigated for different input numbers and pheromone effects. For two inputs (i.e. x, and y coordinates of the nodes), the ACOFIS learned the CFD results of the case for the nanoparticles volume fraction of 0.5. For this condition, the ACOFIS intelligence conditions were not met and the regression number was 0.07. However, increasing the nanoparticles volume fractions as the third input, the number of data increased from 547 to 2685 and as a result, the ACOFIS reached the intelligence with the R value of 0.98. Moreover, the testing different pheromone effect, the value of 0.1 showed the least of RMSE and the best intelligence. The validation test confirmed the high agreement between the CFD results and ACOFIS predictions. The ACOFIS also showed the ability of the accurate pressure and temperature predictions for any other nanoparticle fractions.

Comparing the ANFIS and the ACOFIS, it was shown that both methods could achieve the R and R^2^ of 1 for 3 inputs and the same FIS parameters. The root mean square error of the ACOFIS (~ 1.3) was a little more than that of the ANFIS (~ 0.03), while the total process time of the ANFIS (~ 213 s) was a bit more than that of the ACOFIS (~ 198 s). Although the total time difference was not a lot (less than 15 s), this could increase significantly in real large-scale cases. Finally, it should be noted that the AI algorithms could be so quick prediction (less than 4 min).

This research area is at the beginning. It is recommended to continue the investigations on the other trainers such as neural network, genetic algorithm, bee algorithm, etc., and their tuning parameters for accurate prediction of fluid flow parameters in more complex CFD cases.
